# The assembly and activation of the PANoptosome promote porcine granulosa cell programmed cell death during follicular atresia

**DOI:** 10.1186/s40104-024-01107-3

**Published:** 2024-11-05

**Authors:** Hao Wu, Yingxue Han, Jikang Liu, Rong Zhao, Shizhen Dai, Yajun Guo, Nan Li, Feng Yang, Shenming Zeng

**Affiliations:** 1 National Engineering Laboratory for Animal Breeding, Key Laboratory of Animal Genetics and Breeding of the Ministry of Agriculture, College of Animal Science and Technology, China Agricultural University, Beijing, 100193 China; 2 Department of Clinical Sciences, College of Veterinary Medicine, China Agricultural University, Beijing, 100193 China; 3 College of Animal Science and Technology, Henan Agricultural University, Zhengzhou, 450046 China

**Keywords:** Follicular atresia, Granulosa cells, PANoptosome, Programmed cell death

## Abstract

**Background:**

Follicular atresia significantly impairs female fertility and hastens reproductive senescence. Apoptosis of granulosa cells is the primary cause of follicular atresia. Pyroptosis and necroptosis, as additional forms of programmed cell death, have been reported in mammalian cells. However, the understanding of pyroptosis and necroptosis pathways in granulosa cells during follicular atresia remains unclear. This study explored the effects of programmed cell death in granulosa cells on follicular atresia and the underlying mechanisms.

**Results:**

The results revealed that granulosa cells undergo programmed cell death including apoptosis, pyroptosis, and necroptosis during follicular atresia. For the first time, we identified the formation of a PANoptosome complex in porcine granulosa cells. This complex was initially identified as being composed of ZBP1, RIPK3, and RIPK1, and is recruited through the RHIM domain. Additionally, we demonstrated that caspase-6 is activated and cleaved, interacting with RIPK3 as a component of the PANoptosome. Heat stress may exacerbate the activation of the PANoptosome, leading to programmed cell death in granulosa cells.

**Conclusions:**

Our data identified the formation of a PANoptosome complex that promoted programmed cell death in granulosa cells during the process of follicular atresia. These findings provide new insights into the molecular mechanisms underlying follicular atresia.

**Supplementary Information:**

The online version contains supplementary material available at 10.1186/s40104-024-01107-3.

## Introduction

Folliculogenesis, an ongoing and intricately regulated process throughout an individual’s reproductive lifespan, is marked by significant redundancy, where the number of activated follicles is considerably higher than that of the follicles that ultimately ovulate. Notably, over 99% of these activated follicles naturally undergo atresia [1, 2], which is a complex natural process that acts as the intrinsic regulator of the follicle pool, thus influencing follicular development and encompassing diverse mechanisms of regulated cell death [3].

Changes in the follicular environment are critical in influencing the process of follicular atresia. Variations in hormonal levels, such as reduced follicle stimulating hormone (FSH) and a decrease in luteinizing hormone (LH) pulse amplitude, can hinder the development and survival of follicles [4–6]. Elevated oxidative stress within the follicle often leads to apoptosis of granulosa cells (GCs) and oocyte [7, 8]. Furthermore, disruptions in nutrient availability and blood supply can weaken the follicle’s microenvironment, diminishing its capacity to sustain vital cellular processes [9–11]. These environmental changes collectively accelerate the atresia process, leading to the degeneration of non-dominant follicles and a consequent decline in ovarian reserve. Among these mechanisms, apoptosis of GCs is recognized as the principal initiator of follicular atresia [12–16]. However, recent studies indicate that other forms of GC death, such as necroptosis and pyroptosis, could significantly influence follicular function during follicular atresia [17–20]. These three cell death forms are collectively called programmed cell death (PCD).

PCD in mature organisms is crucial for controlling infection through the elimination intracellular pathogen replication sites and preventing cancer by removing emerging neoplastic cells [21, 22]. Unlike caspase-3-driven apoptosis, necroptosis and pyroptosis are orchestrated by mixed lineage kinase domain-like protein (MLKL) and gasdermin proteins, such as gasdermin D (GSDMD), that form pores in cellular membranes upon activation [23]. Specifically, RIPK3 phosphorylation of MLKL induces significant structural changes and therefore promotes its migration to the plasma membrane and consequent membrane permeabilization [24]. In addition, caspase 1 cleaves GSDMD into N-terminal and C-terminal fragments, with the N-terminal segments assembling into transmembrane pores on the inner cell membrane, disrupting the cytosol-extracellular space boundary [25, 26]. Recent research has characterized the complex interplay and regulatory mechanisms among apoptosis, necroptosis, and pyroptosis.

Recent research advances have introduced the concept of the PANoptosome, a complex that amalgamates critical elements from various cell death pathways [22, 27–30]. The PANoptosome—which was initially identified in response to influenza A virus (IAV) infection—comprises key components such as RIPK1, RIPK3, CASP6, and ZBP1 [22, 31, 32]. Notably, PANoptosome facilitates the simultaneous activation of its constituent pathways and promotes pro-inflammatory cell death. However, the specific role of PANoptosome mediated cell death in GCs and its contribution to follicular atresia remains unclear. In light of this, here we provide evidence that the assembly and activation of the PANoptosome are crucial for the PCD of GCs, contributing to follicular atresia.

## Materials and methods

### Classification of healthy, slightly atretic, and atretic follicles

Ovaries were harvested from 5-month-old, commercially raised Large White gilts at a local abattoir and transported to the laboratory within 2 h in vacuum flasks containing sterile physiological saline at 30–35 °C. Upon arrival, the ovaries were rinsed twice with sterile physiological saline at 37 °C, enriched with 100 IU/L penicillin and 50 mg/L streptomycin.

Gilts alternate between ovaries characterized by small follicles (< 3 mm), known as honeycomb type (HT), and those with medium to large follicles (3–7 mm), referred to as grape type (GT), which are observable on the ovarian surface. Therefore, we focused on GT ovaries during the follicular phase (d 17–21) of the estrous cycle for our study, as follicle selection is believed to originate from the proliferative pool of 1–6 mm follicles present between d 14 and 16 of the estrous cycle. Additionally, during this phase, the atresia rate of small follicles increases, making it easier to assess follicular atresia through morphological observation. The follicles were categorized into healthy (H), slightly atretic (SA), and atretic (A) based on established morphological criteria: Healthy follicles exhibited a vascularized theca interna and clear, amber follicular fluid without debris. In contrast, follicles not meeting these criteria were deemed atretic, with slightly atretic and atretic follicles displaying a gray theca interna and flocculent follicular fluid to varying extents [33, 34].

### Cell culture and experimental design

GCs, harvested from the ovarian follicles of gilts with diameters of 3–5 mm, were pooled and washed twice with phosphate-buffered saline (PBS) (Gibco; 1 mmol/L KH_2_PO_4_, 155 mmol/L NaCl, 3 mmol/L Na_2_HPO_4_·7H_2_O) containing penicillin–streptomycin. The washed GCs were then evenly distributed cultured in 35 mm dishes using Dulbecco’s Modified Eagle Medium (DMEM) supplemented with 10% fetal bovine serum (FBS) and incubated for 24 h at 37 °C in a 5% CO_2_ atmosphere.

**Fig. 1:**
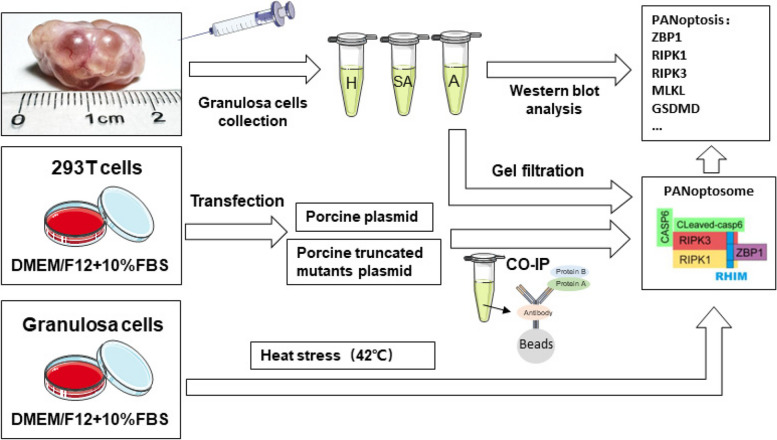
The workflow schematic of this study. Firstly, the levels of programmed cell death marker proteins in the granulosa cells from healthy (H), slightly atretic (SA), and atretic (A) follicles were compared. Next, the interactions of PANoptosome molecules and their interaction domains will be directly studied through vector construction and co-immunoprecipitation techniques. Finally, a heat stress model will be used to study the process of programmed cell death in granulosa cells

Pigs are polyovulatory animals with a high number of ovarian follicles, which facilitates the assessment of follicular health and atresia. Consequently, we selected the porcine ovary as the model system for our study (Fig. 1).

### Western blotting

Samples were incubated in lysis buffer on ice for 10 min. Next, the lysates were transferred into 1.5-mL centrifuge tubes and centrifuged at 12,000 × *g* for 10 min at 4 °C. The protein concentration was determined using a BCA assay kit (TransGen Biotech, Beijing, China). The samples were then prepared for SDS-PAGE by mixing with protein loading buffer (TransGen Biotech, Beijing, China) and boiling at 100 °C for 10 min. Following this, the 30 µg protein samples were subjected to gel electrophoresis at 150 V using a 10% Bis-Tris gel (Epizyme, Shanghai, China) for 1 h. Following electrophoresis, the proteins were transferred onto a nitrocellulose membrane (BioTrace™ NT; Pall Corp., FL, USA) using a wet transfer system and incubated with primary antibodies at 4 °C overnight. The primary antibodies (Table S1) used were diluted 1:1,000 with primary antibody diluent (Huaxing Bio, Beijing, China). After three washes with TBST, the membranes were incubated for 1 h at 37 °C with HRP-bound anti-rabbit IgG and HRP-bound anti-mouse IgG (ZSGB-BIO, Beijing, China). Each antibody was diluted to 1:5,000 with a secondary antibody diluent (Huaxing Bio, Beijing, China).

### Immunofluorescence

Porcine follicles at various stages measuring 3–5 mm in diameter and at various stages, were preserved in 4% phosphate-buffered formaldehyde before being embedded in paraffin. The follicle sections with a thickness of 5 μm were chosen at random and subjected to staining. Immunofluorescence was performed following previously established protocols [35]. For analysis via cellular immunofluorescence, 293T cells were cultured on glass coverslips and fixed with 4% paraformaldehyde for 1 h, then washed with PBS. The cells were permeabilized using 0.5% Triton X-100 for 10 min at 4 °C and blocked with 1% BSA at room temperature for 1 h. Subsequently, the cells were stained with the appropriate primary antibody at 4 °C overnight. Following 3 washes with PBS, the cells were incubated with a secondary antibody diluted in PBS for 1 h at 37 °C, and the nuclei were counterstained with Hoechst 33342 for 5 min. Finally, immunofluorescence samples were analyzed using a confocal microscope (A1HD25, Nikon, Japan), with the images being duly recorded.

### ROS measurement

ROS (reactive oxygen species) levels were measured using the Reactive Oxygen Species Assay Kit (S0033S, Beyotime, Shanghai, China) following the manufacturer’s protocol. This fluorescent method relies on the oxidation of dichlorodihydrofluorescein (DCFH) to dichlorofluorescein (DCF), which emits green fluorescence when excited at 488 nm. The cells were visualized using a confocal microscope (A1HD25, Nikon, Japan).

### Plasmid construction and transfection

The coding sequences of the *ZBP1*, *RIPK3*, and *RIPK1* genes, as well as those of their truncation mutants, were amplified from GC cDNA and cloned into the pcDNA3.1 expression vector (Invitrogen). Accordingly, site-directed mutagenesis was performed as previously described [33]. GFP-Casp6 variants S76A, S76D, S254A, S254D, S76AS254A, and S76DS254D were generated using the Fast Mutagenesis System (FM111; TransGen Biotech). In porcine caspase6, Ser76 and Ser254 were mutated to alanine (caspase6-S76A, caspase6-S254A) and aspartic acid (caspase6-S76D, caspase6-S254D), respectively. 293T cells for transfection were cultured in 60 mm dishes using Dulbecco’s DMEM supplemented with 10% FBS and incubated at 37 °C with 5% CO_2_ until reaching the desired confluency for transfection. 293T cells were transfected with these plasmids using Lipo8000™ (Beyotime, Shanghai, China) in Opti-MEM.

### Molecular docking analysis

To explore intermolecular relationships, we performed rigid protein–protein docking analysis using GRAMM-X (http://gramm.compbio.ku.edu/). The structural domains of RIPK3, RIPK1, ZBP1, and CASP6 were obtained from the AlphaFold Protein Structure Database (https://alphafold.ebi.ac.uk/). We utilized PyMOL (Version 2.4) and PDBePISA (https://www.ebi.ac.uk/pdbe/pisa/) to study protein–protein interactions and conduct visual examinations.

### Co-immunoprecipitation assay

293T cells were lysed in immunoprecipitation buffer (Beyotime, Shanghai, China). The lysates were incubated with 2 μg of specific primary antibodies and gently rotated at 4 °C overnight and then combined with Protein A/G magnetic beads and rotated for 2 h. After 3 washes with IP buffer, the immunoprecipitated samples were eluted and boiled for 10 min in 1% (w/v) SDS sample buffer. Finally, the immunoprecipitated samples were analyzed using Western blot.

### Gel filtration chromatography

GC proteins from porcine atretic follicles were extracted at 4 °C using lysis buffer (20 mmol/L Tris, 150 mmol/L NaCl, 1% Triton X-100), with protease inhibitors added at a ratio of 1/100 of the final volume. Following two rounds of centrifugation at 13,000 × *g* at 4 °C, protein concentration was measured using a BCA protein assay kit. Gel filtration was carried out at 4 °C on a Superose 6 Increase 10/300 GL chromatography column (Cytiva, USA), equilibrated with PBS buffer (10 mmol/L phosphate buffer, 140 mmol/L NaCl, pH 7.4) at a flow rate of 0.5 mL/min. Fractions of 0.5 mL were collected, and 30 μL from each fraction was analyzed by Western blotting.

### Heat stress exposure

GCs were cultured in an incubator (Thermo Fisher Scientific) at 43 °C with humidified 5% CO_2_ for 4 h, followed by incubation at 37 °C for the specified durations. HSP70 expression increases in response to stress, serving as a reliable biomarker for heat stress [36, 37].

### Data availability

The proteome data for porcine GCs from healthy, slightly atretic, and atretic follicles were obtained from DOI: 10.3389/fcell.2020.624985.

### Statistical analysis

All statistical analyses were performed using SPSS 16.0 software (IBM, USA). Data are presented as mean ± SD. Differences between groups were assessed using ANOVA, followed by an LSD post hoc test. *P* < 0.05 was considered statistically significant.

## Results

### PCD is activated in GCs during porcine follicular atresia

**Fig. 2:**
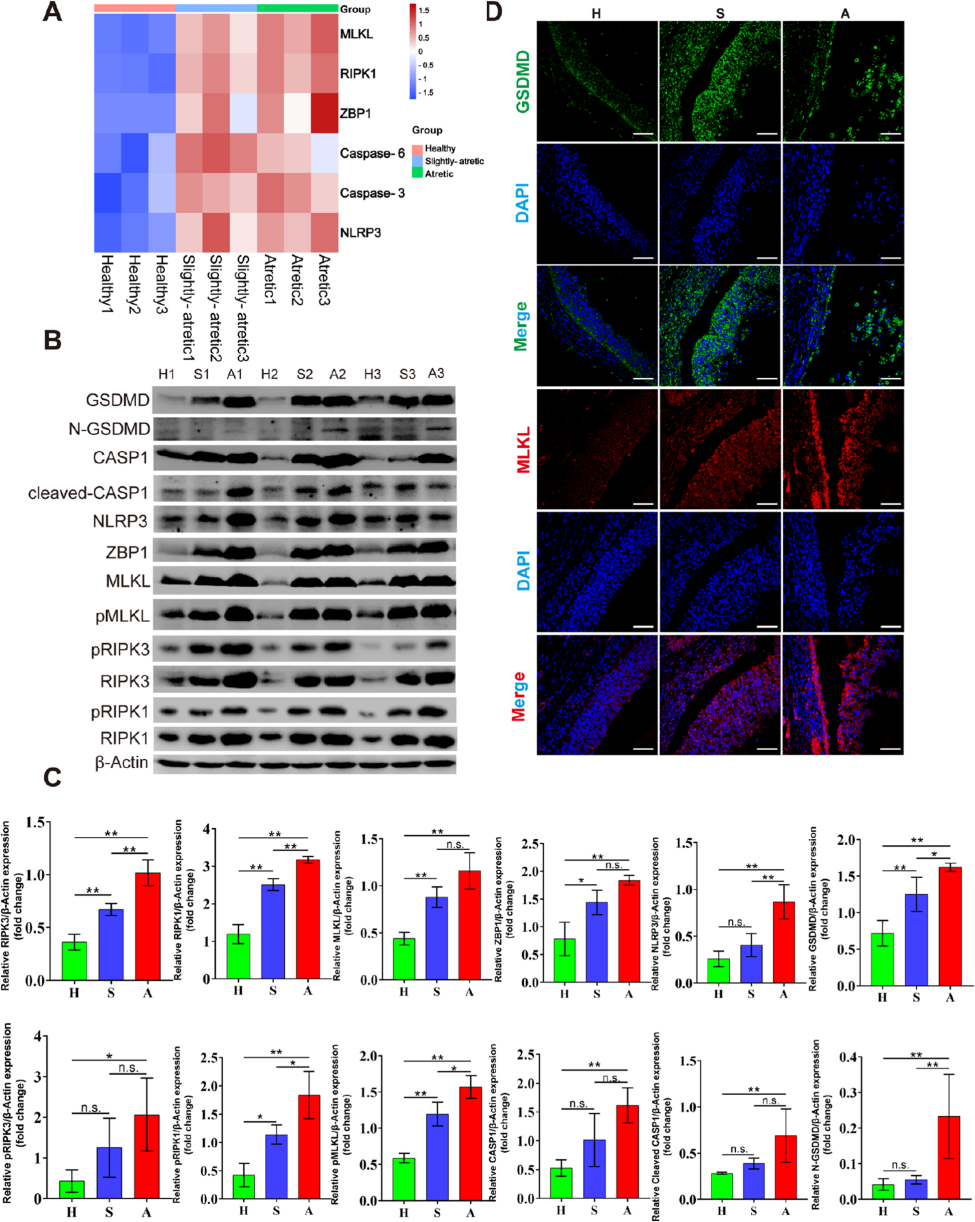
PCD is activated in GCs during porcine follicular atresia. **A** Heatmap of differentially regulated transcripts enriched in PCD-related genes between healthy and atretic follicular GCs. **B** Immunoblots of GC lysates from healthy (H), slight atretic (S), and atretic (A) in porcine follicles. **C** Quantitative analysis of protein levels in **B**. **D** Immunofluorescence staining of GSDMD and MLKL in H, S and A porcine follicles. The scale bar represents 100 μm. Nuclei were stained with DAPI (blue). The results are presented as mean ± SD. ^*^P < 0.05; ^**^P < 0.01; n.s., not significant

To explore the involvement of PCD in porcine GCs during follicular atresia, we analyzed differential gene expression using an existing proteome dataset from GCs comprising healthy, slightly atresic, and atretic follicles. Figure 2A illustrates a heatmap that highlights the specific gene regulation within the PCD pathway, showing changes in protein levels of key genes in GCs from atretic follicles. Further investigation through Western blot analysis identified the presence of PCD markers in GCs from healthy, slightly atretic, and atretic follicle (Fig. 2B). Compared to healthy cells, those from atretic follicles demonstrated notable increases in the expression of necroptosis markers (RIPK1, RIPK3, and MLKL) and enhanced phosphorylation (*P* < 0.01, Fig. 2C). Additionally, the expression levels of pyroptosis markers (NLRP3, CASP1, and GSDMD) were increased, with CASP1 and GSDMD showing signs of cleavage in the atretic categories (*P* < 0.01, Fig. 2C). Immunofluorescence imaging (Fig. 2D) revealed the cytoplasmic localization and expression patterns of GSDMD and MLKL in GCs, where the intensity of the staining was correlated with the progression of follicular atresia. These findings indicate that GCs may undergo both pyroptosis and necroptosis during follicular atresia.

### PANoptosome molecules directly interact

**Fig. 3:**
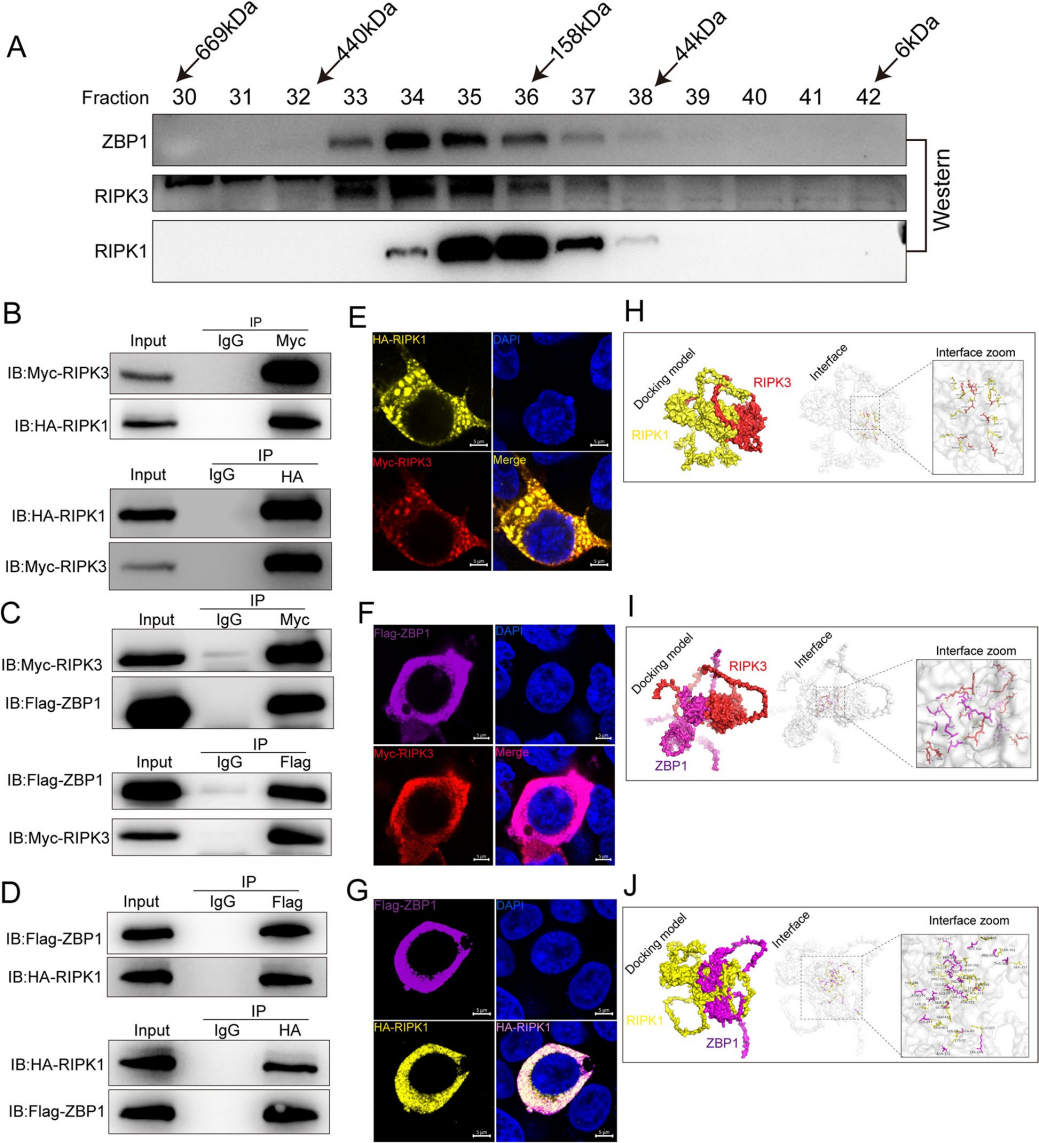
PANoptosome molecules directly interact. **A** Gel filtration analysis of the PANoptosome complex. The Western blots of column fractions were performed using the indicated antibodies.** B** HA-RIPK1 and Myc-RIPK3 were co-transfected into 293T cells for 48 h for immunoprecipitation. **C** Flag-ZBP1 and Myc-RIPK3 were co-transfected into 293T cells for 48 h for immunoprecipitation. **D** Flag-ZBP1 and HA-RIPK1 were co-transfected into 293T cells for 48 h for immunoprecipitation. **E** Co-transfection of HA-RIPK1 and Myc-RIPK3 in 293T cells, showing DNA stained with DAPI. Different tag proteins were stained with mouse- and rabbit-derived primary and secondary antibodies. Scale bar: 5 = μm. **F** Co-transfection of Myc-RIPK3 and Flag-ZBP1 in 293T cells, showing DNA stained with DAPI. Different tag proteins were stained with mouse- and rabbit-derived primary and secondary antibodies. Scale bar: 5 = μm. **G** Co-transfection of HA-RIPK1 and Flag-ZBP1 in 293T cells, showing DNA stained with DAPI. Different tag proteins were stained with mouse- and rabbit-derived primary and secondary antibodies. Scale bar: 5 = μm. **H** Interfacing residues between porcine RIPK3 and RIPK1 proteins (RIPK3, red; RIPK1, yellow; hydrogen bond interaction indicated by dotted lines). **I** Interfacing residues between porcine RIPK3 and ZBP1 proteins (RIPK3, red; ZBP1, purple; hydrogen bond interaction are indicated by dotted lines). **J** Interfacing residues between porcine RIPK1 and ZBP1 proteins (RIPK1, yellow; ZBP1, purple; hydrogen bond interactions are indicated by dotted lines)

We conducted gel filtration experiments to confirm that the core PANoptosome proteins, RIPK3, RIPK1, and ZBP1, form a complex (Fig. 3A). To evaluate the interaction between the components of the cell death complex directly, we constructed plasmids encoding porcine RIPK3, RIPK1, and ZBP1 for transfection into 293T cells, as the endogenous proteins in GCs cannot be pulled down with currently available antibodies. Subsequent experiments demonstrated reciprocal co-immunoprecipitation between RIPK3 and both RIPK1 and ZBP1, as illustrated in Fig. 3B and C. Additionally, RIPK1 and ZBP1 exhibited mutual co-immunoprecipitation (Fig. 3D). Immunofluorescence analysis revealed co-localization of RIPK3, RIPK1, and ZBP1 (Fig. 3E–G). Additionally, bioinformatic analysis demonstrated the formation of hydrogen bonds between specific amino acid residues of these proteins, suggesting a stable docking model (Fig. 3H–I). Collectively, the results support the hypothesis that these porcine proteins interact directly to form a PANoptosome complex.

### RIPK3, RIPK1, and ZBP1 interact via the RHIM domain

**Fig. 4:**
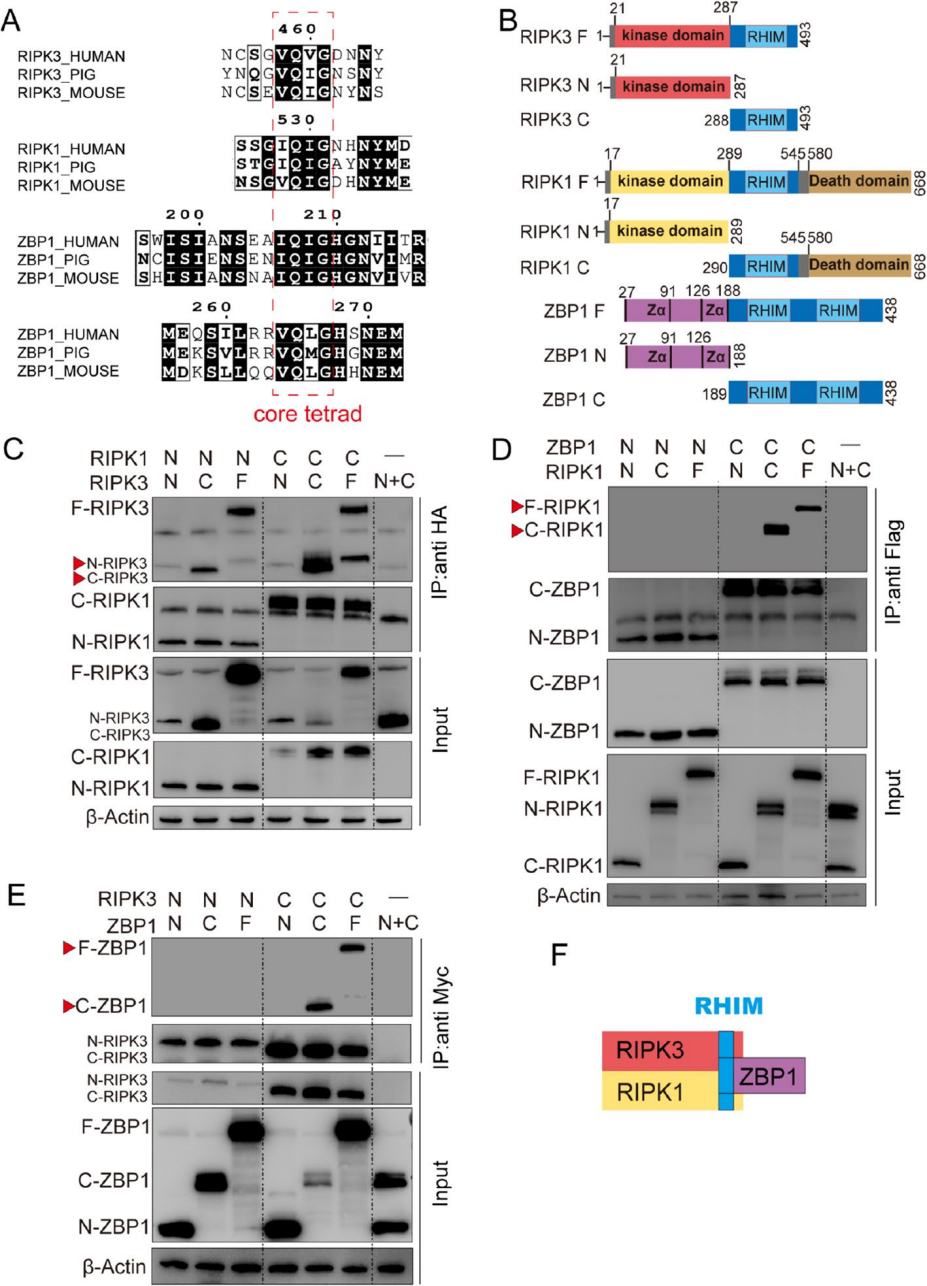
RIPK3, RIPK1, and ZBP1 interact via the RHIM domain. **A** Amino acid sequences of RHIM-containing regions of human, pig, and mouse proteins RIPK1, RIPK3, and ZBP1. **B** Schematic representation of domains in porcine CASP6, RIPK1, and RIPK3 in full-length (F), N-terminal (N), and C-terminal (C) constructs. **C** Immunoprecipitates and total lysates were obtained from 293T cells following co-transfection with the specified variants of RIPK3 and RIPK1 for a duration of 48 h. **D** Immunoprecipitates and total lysates were obtained from 293T cells following co-transfection with the specified variants of RIPK3 and ZBP1 for a duration of 48 h. **E** Immunoprecipitates and total lysates were obtained from 293T cells following co-transfection with the specified variants of RIPK1 and ZBP1 for a duration of 48 h. **F** Diagram of the interaction mode of RIPK3, RIPK1, and ZBP1

We examined the interaction domains of RIPK3, RIPK1, and ZBP1. A comparative analysis of RIPK3, RIPK1, and ZBP1 amino acid sequences across porcine, mouse, and human species revealed the presence of a (V/I)-Q-(V/I/L/C)-G motif—characteristic of the RIP homotypic interaction motif (RHIM)—at the C-termini of all three proteins (Fig. 4A). To further detemine whether the interactions between these proteins depended on the RHIM domain, truncated mutants of RIPK3, RIPK1, and ZBP1 were generated in 293T cells to map the essential interaction domains (Fig. 4B). Both the amino-terminal and carboxy-terminal domains of RIPK1 interacted with the corresponding domains of RIPK3 (Fig. 4C). The carboxyl termini of these two proteins exhibited strong binding (Fig. 4C, indicated by the red arrows). Interestingly, deficiency in RHIM prevented ZBP1–RIPK3 binding as well as RIPK1 binding (Fig. 4D), implying the crucial role of the RHIM domain in mediating protein interactions.

### Caspase-6 is a component of the PANoptosome in GCs

**Fig. 5:**
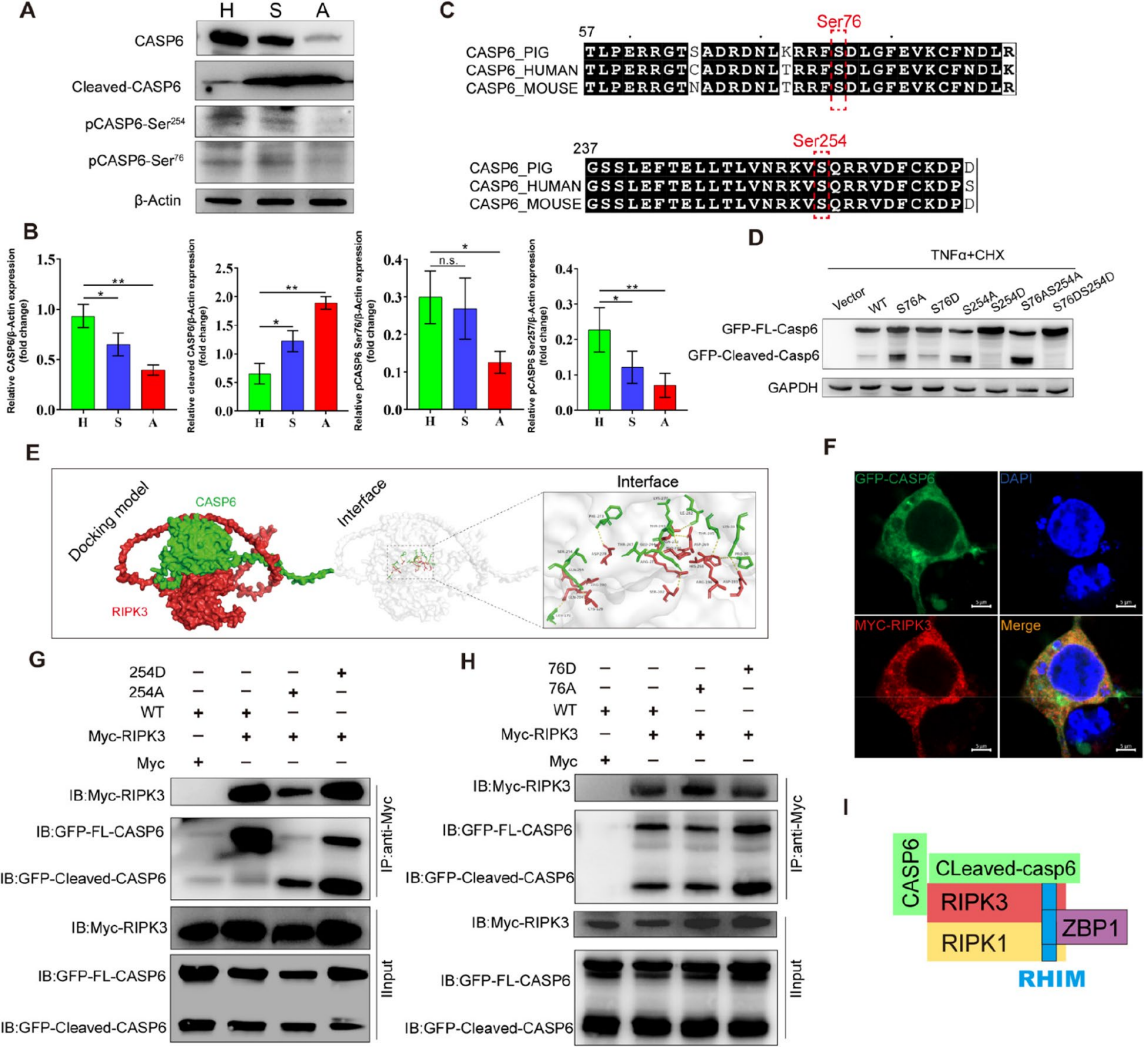
Caspase-6 as a component of the PANoptosome in GCs. **A** Immunoblot of GC lysates from healthy (H), slight atretic (S), and artretic (A) follicles. **B** A quantitative analysis of the protein levels shown in **A**. **C** Alignment of the amino acid sequences surrounding Ser257 and Ser76 of caspase-6 in humans, mice, and pigs. **D** 293T cells overexpressing GFP-caspase-6 wild type, S76A, S76D, S254A, S254D, S76AS254A, or S76DS254D mutants were treated with 10 μg/ml CHX and 25 ng/ml TNFα for 2 h. The immunoblot analysis of cell lysates is shown. (Single-letter abbreviations for amino acid residues are as follows: A for Ala, D for Asp, and S for Ser. In the case of the mutants, specific amino acid substitutions occurred at defined positions. For examples, S76A signifies the replacement of serine at position 76 with alanine.) **E** Surface diagram of the docking model and the interfacing residues between CASP6 and RIPK3 proteins (CASP6, green; RIPK3, red; hydrogen bond interactions are indicated by dotted lines). **F** Co-transfection of GFP-CASP6 and Myc-RIPK3 in 293T cells is shown. DNA was stained with DAPI. Scale bar, 5 μm. **G** Co-immunoprecipitation assay of the interactions between RIPK3 and caspase-6 or its mutants at site 254. **H** Co-immunoprecipitation assay to assess the interactions between RIPK3 and caspase-6 or its mutants at site 76. **I** Caspase-6 as a schematic diagram of the PANoptosome structure. The results are presented as the means ± SD. ^*^P < 0.05; ^**^P < 0.01; n.s., not significant

To explore the mechanistic role of caspase-6 in GCs during the process of follicular atresia, we initially assessed the variations in caspase-6 expression levels among GCs from follicles in different states. Caspase-6 undergoes cleavage in the GCs of atretic follicles, accompanied by a decrease in phosphorylation trends at its Ser76 and Ser254 sites (*P* < 0.01, Fig. 5A). Both Ser254 and Ser76, along with their respective adjacent sequences in caspase-6, demonstrated conservation across diverse species (Fig. 5B). Moreover, wild-type caspase-6, the non-phospho-mimetic Ser76, S254A mutant, and the phospho-mimetic S76D, S254D mutants were overexpressed in 293T cells. Following this, the cells underwent treatment with low doses of TNFα and CHX to induce the cleavage of caspase-6. The S254A, S76A, and S254AS76A mutants exhibited increased sensitivity to cleavage, whereas the S254D, S76D, and S76DS254D mutants displayed complete resistance (Fig. 5D).

To investigate whether caspase-6 contributes to the formation of the PANoptosome complex, we performed a protein–protein docking analysis between caspase-6 and RIPK3. The analysis revealed the formation of hydrogen bonds at specific amino acid residue sites, which indicated the establishment of a robust and stable protein docking model between caspase-6 and RIPK3 (Fig. 5E). Subsequently, we co-overexpressed caspase-6 along with RIPK1, ZBP1, and RIPK3 in 293T cells, enabling us to evaluate the interaction between caspase-6 and the components of the complex. Additionally, immunoprecipitation assays demonstrated the co-immunoprecipitation of caspase-6 and its mutant with RIPK3. Notably, both the full-length and cleaved forms of caspase-6 could interact with RIPK3 (Fig. 5G and H). Immunofluorescence co-localization signals further supported the interaction between caspase-6 and RIPK3 (Fig. 5F). However, we did not observe the binding of caspase-6 to either ZBP1 or RIPK1 (Fig. S1), suggesting a potential recruitment mechanism of caspase-6 to the complex via RIPK3 (Fig. 5I and S2).

### Heat stress triggers GC pyroptosis and necroptosis

**Fig. 6:**
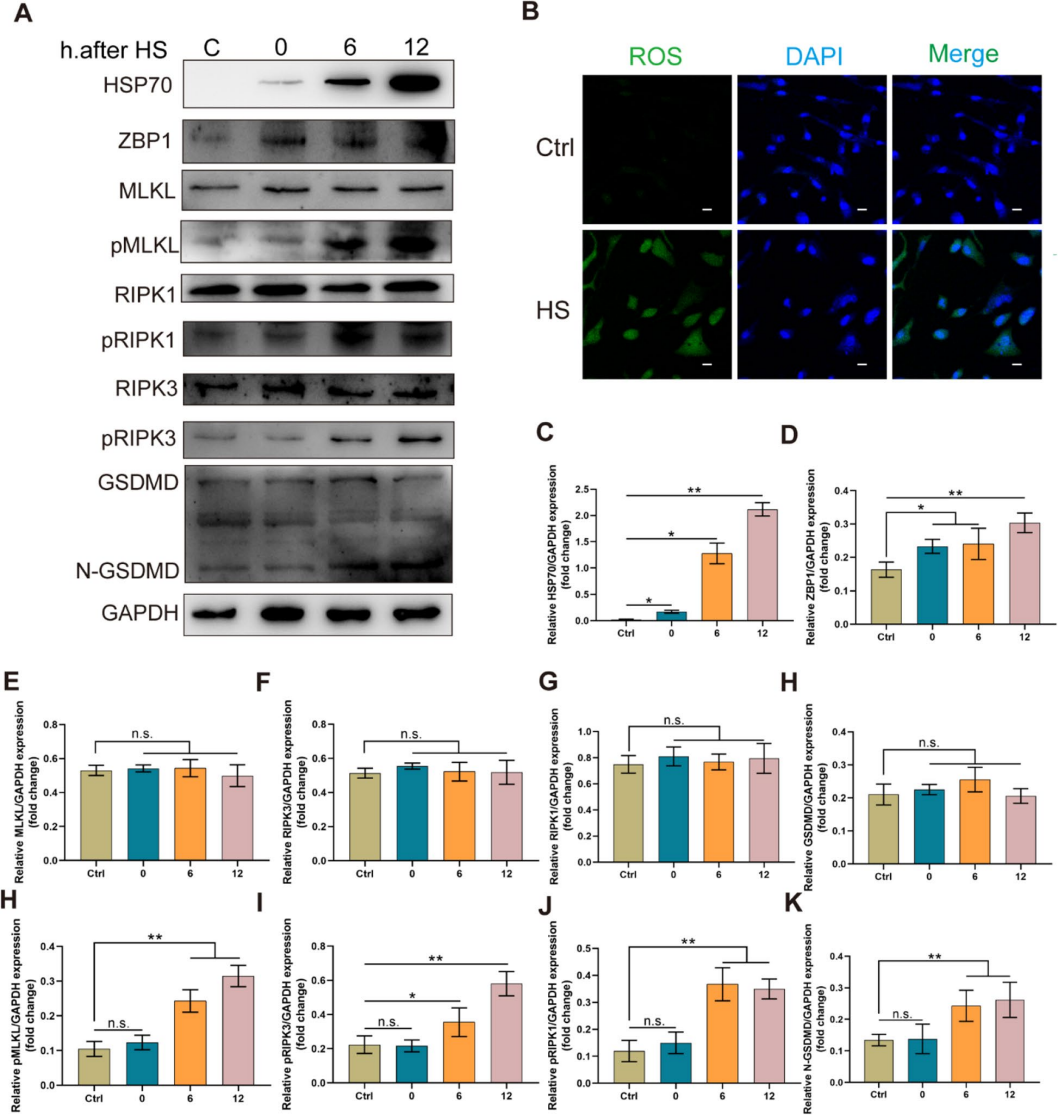
Heat stress triggers granulosa cell pyroptosis and necroptosis. **A** Western blot analysis of the quantity of indicated proteins in GCs at the indicated time points after heat stress (43 °C for 4 h). **B** Fluorescent images showing ROS staining in control and heat stress-exposed GCs. Scale bar: 10 μm. **C–K** Quantitative analysis of protein levels in **A**. The results are presented as mean ± SD. ^*^P < 0.05; ^**^P < 0.01; n.s., not significant

To investigate the effect of heat stress on GC PCD, cultured GCs were exposed to 43 °C for 4 h. This exposure significantly increased the phosphorylation levels of RIPK3, RIPK1, and MLKL proteins, as well as the cleavage of GSDMD, within 12 h post-heat stress. (*P* < 0.01, Fig. 6A, C–K). Furthermore, these cells demonstrated an elevated accumulation of intracellular reactive oxygen species (ROS) compared to the controls following heat stress (Fig. 6B).

## Discussion

**Fig. 7:**
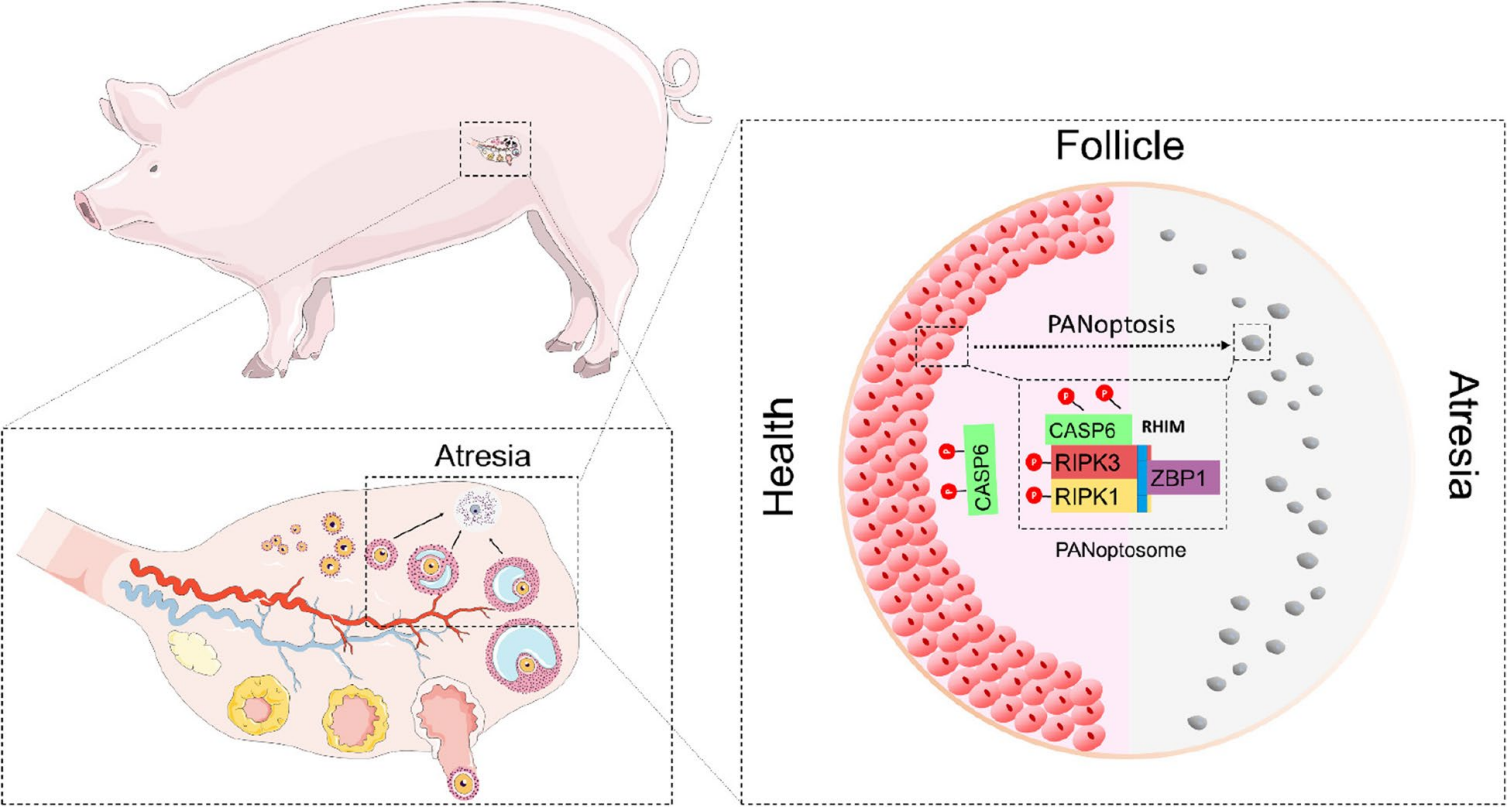
The mechanism of PANoptosome formation and regulation of granulosa cell programmed cell death during follicular atresia

Follicular development and ovulation are key determinants of the reproductive potential of sows. However, the onset of follicular atresia and degeneration, triggered by the death of GCs, is a limiting factor for sow fertility. These intricate processes of follicular atresia and GC death are tightly regulated by a diverse array of factors. In the present study, we discovered that GCs undergo not only undergo apoptosis but also pyroptosis and necroptosis during the process of follicular atresia. Furthermore, we identified the assembly and activation of a PANoptosome during follicular atresia that mediates the occurrence of these three types of cell death (Fig. 7). Our results provide new insights into the mechanisms of follicular atresia and GC PCD.

Apoptosis is the primary mechanism underlying GC death processes during follicle atresia [3]. Beyond apoptosis, necroptosis has been observed in GCs within the ovary as a response to the accumulation of reactive oxygen species (ROS). In vitro studies have shown that subjecting human GCs to serum starvation leads to the generation of ROS, triggering both necroptosis and apoptosis [38]. Notably, mediators of necroptosis, such as phosphorylated MLKL, RIPK1, and RIPK3 proteins were identifiable within the GCs of both pre-antral and antral follicles in macaque ovaries, as well as in the corpora lutea of human ovaries [39, 40]. Furthermore, recent research has demonstrated the upregulation of gene expression of *RIPK1* and *RIPK3* in atretic bovine follicles, in contrast to healthy follicles [18]. Notably, oxidative stress can prime the NLRP3 inflammasome, leading to caspase-1 activation and pyroptosis [41, 42]. Meanwhile, a recent study involving dairy cows revealed that culturing GCs with non-esterified fatty acids induced oxidative stress, pyroptosis, and inflammation [19]. Consistent with these results, we observed an upregulation of genes related to pyroptosis and necroptosis, including *ZBP1*, *RIPK3*, *RIPK1*, and *MLKL*, as well as their phosphorylated forms. Additionally, cleavage of GSDMD and caspase-1 was occurred, indicating the occurrence of pyroptosis and necroptosis in GCs during follicle atresia. This suggests a potential contribution of GC PCD to follicular atresia.

Interestingly, it has been found that the above proteins can act as integral components of larger cell death-inducing complexes known as PANoptosomes, which integrate components from multiple cell death pathways and drive a form of lytic, innate immune inflammatory cell death termed PANoptosis [21, 43, 44]. Previous studies have suggested that a singular cell death complex may regulate the activation of these pathways by orchestrating interactions among their key components [30, 45]. Accordingly, to verify PANoptosome formation in GCs during follicular atresia, we generated porcine RIPK3, RIPK1, and ZBP1 plasmids and conducted immunoprecipitation experiments. Our findings demonstrated interactions among RIPK1, RIPK3, and ZBP1, suggesting the assemble of a PANoptosome within the GCs. This aligns with previous observations demonstrating that macrophages assemble and activate the PANoptosome to induce PANoptosis in response to invasion by pathogenic microorganisms [30]. Nevertheless, the composition of PANoptosomes has varied among studies, and the phenotypes appear to depend on the stimulus provided [45].

Homotypic interactions are essential in various signaling complexes. While the compositions of individual PANoptosome may differ, each contains components with key domains such as RHIMs that are crucial for assembly [29]. The RHIM domains in these proteins include a core tetrad characterized by the consensus sequence (V/I)-Q-(V/I/L/C)-G that is essential for intermolecular protein interactions and signal transduction [22, 43, 46]. Specifically, RIPK3-dependent signal transduction relies heavily on the precise assembly of RHIM complexes. Thus, any substitution mutants in the RHIM domain residues can significantly disrupt downstream signaling events [47]. For example, altering a single valine residue within the RIPK3 core tetrad can prevent necroptosis in vitro [48]. In mouse bone marrow-derived macrophages, a lack of RHIM impeded the binding between ZBP1 and RIPK1 [49]. Consistent with these findings, our study demonstrated that ZBP1 engages RIPK3 and RIPK1 through the receptor interacting protein RHIM, and in the absence of the RHIM structure of ZBP1 cannot interact with RIPK3 or RIPK1, suggesting that RHIM is essential for the assembly of a PANoptosome during porcine follicle atresia.

Caspase-6 has long been regarded as a mysterious protein. In colorectal adenocarcinoma cells, phosphorylation of the Ser254 of caspase-6 by AMPK related protein kinase 5 has been identified [50]. This phosphorylation event has been shown to suppress both the cleavage and activation of caspase-6 [51]. Our previous proteomic phosphorylation studies have shown that the Ser76 site is significantly phosphorylated in GCs of healthy follicles, with dephosphorylation occurring during the atresia process. To validate this phosphorylation event, we generated a polyclonal antibody against the phospho-S76 peptide. Consistent with previous studies, we observed that the phosphorylation of caspase-6 at the Ser76 and Ser254 site in GCs gradually decreases, thereby leading to its cleavage during follicular atresia (Fig. 4A). Ser76 serves as another active site of caspase-6. Dephosphorylation at this site facilitates the cleavage and activation of caspase-6, ultimately leading to apoptosis [33]. Despite being classified as an apoptotic executor caspase and being implicated in various diseases such as nonalcoholic steatohepatitis, the full extent of caspase’s functional significance and its involvement in other biological processes remain unclear [52]. Recent studies have suggested that caspase-6 plays a key role in innate immunity and the activation of the ZBP1-NLRP3 inflammasome [22, 53, 54]. Notably, observations suggest a scaffolding function for caspase-6, as evidenced by the enhanced interaction between RIPK3 and ZBP1 in the presence of catalytically inactive or uncleavable caspase-6 mutants, particularly during infections with pathogens such as IAV [22]. In the present study, we observed that caspase-6 was a component of the PANoptosome. Interestingly, both the full-length and cleaved forms of caspase-6 interacted with RIPK3 but showed no interaction with RIPK1 or ZBP1. This observation suggests that the function of caspase-6 depends on the RIPK3 pathway.

The activation of the PANoptosome integrates multiple programmed cell death pathways, providing GCs with a flexible mechanism to adapt to stress, efficiently clear damaged cells, and protect the follicular microenvironment. Pyroptosis and necroptosis promote inflammatory responses and coordinate cell death with immune responses, making the PANoptosome more effective at eliminating GCs during follicular atresia compared to a single cell death pathway, thereby ensuring proper ovarian function. Different from apoptotic bodies formed by apoptosis, the most prominent feature of pyrodeath and necroptosis is the formation of pores in the cell membrane [26, 55]. This process results in the release of pro-inflammatory cytokines, particularly IL-1β and IL-18. The release of these cytokines, along with other intracellular contents like DAMPs, into the extracellular space triggers a strong inflammatory response. This inflammation recruits immune cells to the site of infection or damage, amplifies the immune response, and promotes the clearance of pathogens or damaged cells. It also provides evidence for the increase in macrophages in follicles during follicular atresia [56–58]. Therefore, further experiments are needed to prove the role of pyroptosis and necroptosis in follicular atresia.

The activation mechanisms of the PANoptosome in follicular atresia of GCs are currently unclear. In previous studies, the activation and assembly of the PANoptosome depended on various pathogen-induced innate immune responses [21, 22, 27]. Recent research has demonstrated that heat stress can induce PANoptosis [59]. Furthermore, under heat stress, the generated ROS can also induce necroptosis and pyroptosis [59–63]. A recent study has shown that heat stress can trigger necroptosis, apoptosis, and pyroptosis via the ZBP1-RIPK3 signaling pathway. In addition, the genetic deletion or targeted inhibition of ZBP1, RIPK3, or both caspase-8 and MLKL can prevent these pathways of cell death, thereby improving cell survival [59]. GCs exposed to heat stress in vitro demonstrated impaired physiological functions, including increasing intracellular accumulation of ROS-inducing apoptosis, and reduced synthesis of estradiol and progesterone [36, 37]. The above results indicate that cells may undergo apoptosis, necroptosis, and pyroptosis under heat stress and oxidative stress. Our previous studies have shown that heat stress induces apoptosis in porcine GCs [13]. In the present study, we found that heat stress could activate the PANoptosome, promoting necroptosis and pyroptosis in porcine GCs. Additionally, intracellular ROS levels also increased, which suggested a mechanistic connection between ROS production and PANoptosis. These results provide new insights into how heat waves during the summer cause follicular atresia, leading to reduced reproductive capacity in livestock [36, 37, 64].

As a critical determinant of follicular fate, FSH promotes the proliferation and differentiation of GCs. However, whether it also inhibits the activation of the PANoptosome in GCs as a protective mechanism for follicles requires further investigation. The ovarian follicular vascular system plays a vital role in supplying the essential nutrients, oxygen, and hormones necessary for follicle development [65]. Angiogenesis was active and increasing in selected follicles, while those undergoing atresia exhibited minimal blood vessel development [10, 66]. In atretic follicles, the reduction in blood vessel density within the basal membrane creates a hypoxic environment in GCs, leading to apoptosis [67]. Recent studies have shown that hypoxic conditions can simultaneously induce pyroptosis and necroptosis in mammalian cells [68–70]. The hypoxic environment within the follicle is a potential detrimental factor that triggers the PANoptosome in GCs, leading to PANoptosis. Furthermore, ovarian aging is often accompanied by an increase in follicular atresia and a decrease in follicle count [71]. In addition to apoptosis, pyroptosis and necrosis also play a role in the process of ovarian aging [72, 73]. Recent studies have shown that nicotinamide mononucleotide and spermidine can improve the quality of mouse oocytes, thereby delaying aging [74, 75]. However, these studies did not investigate the mechanisms underlying their effects on GCs. It is possible that they inhibit PANoptosis in GCs, thereby enhancing the communication between GCs and oocytes. This will be a key focus of future research.

## Conclusions

Our study revealed that GCs undergo PANoptosis during follicular atresia. By demonstrating the interaction among RIPK1, RIPK3, ZBP1, and CASP6, we provide evidence for the existence of a cell death complex, the PANoptosome, which orchestrates various pathways in GCs. This PANoptosome likely plays a crucial role in regulating follicular atresia (Fig. 7). As such, understanding the molecular events underlying follicular atresia is crucial for elucidating the physiological processes involved in ovarian follicle development and dynamics.

## Supplementary Information

**Additional file 1** **Table S1** List of primary antibodies used for western blot analysis. **Table S2** Prediction of molecular docking at hydrogen bond binding sites for RIPK3 and RIPK1. **Table S3** Prediction of molecular docking at hydrogen bond binding sites for ZBP1 and RIPK3. **Table S4** Predictionof molecular docking at hydrogen bond binding sites for RIPK1 and ZBP1. **Table S5** Prediction of molecular docking at hydrogen bond binding sites for RIPK3 and CASP6.

**Additional file 2** **Fig. S1** CASP6 cannot interact with RIPK1 and ZBP1.**Fig. S2** CASP6 as a component of the PANoptosome.

## Data Availability

The proteome data of porcine GCs from healthy, slightly atresia and atretic follicles was downloaded from doi: 10.3389/fcell.2020.624985.

## Abbreviations

A: Atretic
FSH: Follicle stimulating hormone
GC: Granulosa cell
GSDMD: Gasdermin D
H: Healthy
IAV: Influenza A virus
MLKL: Mixed lineage kinase domain-like protein
PBS: Phosphate buffered saline
PCD: Programmed cell death
RHIM: RIP homotypic interaction motif
ROS: Reactive oxygen species
SA: Slightly atretic
