# Supplementary material for: The assembly and activation of the PANoptosome promote porcine granulosa cell programmed cell death during follicular atresia
Source: J Anim Sci Biotechnol. 2024 Nov 5;15:147. doi: 10.1186/s40104-024-01107-3 (PMC11536665; doi:10.1186/s40104-024-01107-3)
Supplement: Supplementary file 1 — Additional file 1 Table S1 List of primary antibodies used for western blot analysis. Table S2 Prediction of molecular docking at hydrogen bond binding sites for RIPK3 and RIPK1. Table S3 Prediction of molecular docking at hydrogen bond binding sites for ZBP1 and RIPK3. Table S4 Predictionof molecular docking at hydrogen bond binding sites for RIPK1 and ZBP1. Table S5 Prediction of molecular docking at hydrogen bond binding sites for RIPK3 and CASP6. [file 40104_2024_1107_MOESM1_ESM.docx]

**Additional file 1**

**Table S1** List of primary antibodies used for western blot analysis

**Table S2** Prediction of molecular docking at hydrogen bond binding sites for RIPK3 and RIPK1

**Table S3** Prediction of molecular docking at hydrogen bond binding sites for ZBP1 and RIPK3

**Table S4** Prediction of molecular docking at hydrogen bond binding sites for RIPK1 and ZBP1

**Table S5** Prediction of molecular docking at hydrogen bond binding sites for RIPK3 and CASP6

**Table S1** List of primary antibodies used for western blot analysis

| **Antibody** | **Source** | **Catalog code** |
| --- | --- | --- |
| GSDMD | Affinity Biosciences | AF4012 |
| CASP1 | Affinity Biosciences | AF5418 |
| RIPK3 | Affinity Biosciences | DF10141 |
| RIPK1 | Proteintech | 17519-1-AP |
| pRIPK1 | Proteintech | 28252-1-AP |
| MLKL | Proteintech | 21066-1-AP |
| pRIPK3 | Abcam | ab195117 |
| Myc | Proteintech | 60003-2-Ig |
| HA | Proteintech | 81290-1-RR |
| Flag | Proteintech | 20543-1-AP |
| GFP | Proteintech | 50430-2-AP |
| pMLKL | Cell Signaling Technology | 91689S |
| ZBP1 | BIOSS | bs-13559R |
| NLRP3 | Abmart | TD7438 |
| HSP70 | Stress Gen Biotechnologies | N27F3-4 |
| CASP6 | Abcam | ab185645 |
| pCASP6 | Thermo | PA5-38241 |
| GAPDH | Proteintech | 60004-1-Ig |
| β-ACTIN | Absin | abs132001 |

**Table S2** Prediction of molecular docking at hydrogen bond binding sites for RIPK3 and RIPK1

|  | **RIPK3** | **Dist.[A]** | **RIPK1** |
| --- | --- | --- | --- |
| 1 | C: HIS 237[ ND1] | 2.25 | A: ARG  69[ O] |
| 2 | C: GLN 247[ NE2] | 2.56 | A: LYS  37[ O] |
| 3 | C: GLY 249[ N] | 3.19 | A: GLN 319[ OE1] |
| 4 | C: GLN 263[ NE2] | 3.15 | A: GLU  93[ OE2] |
| 5 | C: SER 267[ OG] | 2.39 | A: ARG  71[ O] |
| 6 | C: HIS 268[ N] | 2.52 | A: ARG  71[ O] |
| 7 | C:ASP 278[ N] | 3.80 | A: GLU 294[ OE1] |
| 8 | C: ASN 282[ N] | 3.38 | A: GLU 298[ OE1] |
| 9 | C: ASN 282[ N] | 3.43 | A: GLU 298[ OE2] |
| 10 | C: LYS 292[ N] | 3.74 | A: LYS 302[ O] |
| 11 | C: ASN 272[ O] | 2.63 | A: SER  73[ OG] |
| 12 | C:ASP 278[ OD1] | 3.84 | A: TYR 289[ OH] |
| 13 | C:ASP 278[ OD2] | 3.67 | A: ARG 286[ NH1] |

**Table S3** Prediction of molecular docking at hydrogen bond binding sites for ZBP1 and RIPK3

|  | **ZBP1** | **Dist.[A]** | **RIPK3** |
| --- | --- | --- | --- |
| 1 | C: LYS  62[ NZ] | 3.81 | A: SER 146[ OG] |
| 2 | C: THR  24[ OG1] | 3.21 | A: SER 172[ OG] |
| 3 | C: THR  24[ OG1] | 3.29 | A: ASN 195[ OD1] |
| 4 | C: LYS  72[ NZ] | 3.48 | A: GLN 225[ OE1] |
| 5 | C: ASN 182[ ND2] | 2.71 | A: GLU 231[ OE1] |
| 6 | C: ASN 164[ ND2] | 3.14 | A: ARG 236[ O] |
| 7 | C: ASN 164[ ND2] | 3.57 | A: HIS 237[ O] |
| 8 | C: MET 170[ N] | 2.68 | A:ASP 243[ OD2] |
| 9 | C: THR 176[ OG1] | 3.16 | A: TYR 467[ O] |
| 10 | C: GLN 188[ N] | 3.82 | A: TYR 467[ O] |
| 11 | C:ASP  30[ OD2] | 3.89 | A: GLU 178[ N] |
| 12 | C:ASP  30[ OD2] | 3.48 | A: SER 179[ N] |
| 13 | C: GLN  58[ O] | 2.97 | A: LYS  29[ NZ] |
| 14 | C: GLN  66[ OE1] | 2.58 | A: CYS 181[ N] |
| 15 | C: GLN  70[ OE1] | 3.40 | A: ARG 173[ NH2] |
| 16 | C: GLY  83[ O] | 3.84 | A: GLN 225[ NE2] |
| 17 | C: ARG 165[ O] | 3.58 | A: LEU 242[ N] |
| 18 | C: ASN 172[ O] | 2.40 | A: ASN 466[ ND2] |
| 19 | C:ASP 178[ O] | 3.47 | A: ARG 236[ NH1] |
| 20 | C: GLN 179[ O] | 2.85 | A: ARG 236[ NH2] |
| 21 | C: GLU 277[ OE1] | 2.66 | A: SER 452[ OG] |

**Table S4** Prediction of molecular docking at hydrogen bond binding sites for RIPK1 and ZBP1

|  | **RIPK1** | **Dist.[A]** | **ZBP1** |
| --- | --- | --- | --- |
| 1 | C: SER 335[ N] | 2.78 | A: GLU 104[ OE1] |
| 2 | C: LYS  37[ NZ] | 3.86 | A: ASN 172[ OD1] |
| 3 | C: LYS  30[ NZ] | 3.31 | A: GLN 202[ O] |
| 4 | C: THR 180[ N] | 3.42 | A: ASN 204[ OD1] |
| 5 | C: ARG 390[ NH2] | 3.33 | A: LEU 214[ O] |
| 6 | C: ALA 313[ N] | 3.80 | A: ASN 222[ OD1] |
| 7 | C: ASN 312[ N] | 2.49 | A: ASN 222[ OD1] |
| 8 | C: ARG 102[ NH2] | 3.21 | A: SER 223[ O] |
| 9 | C: GLY  98[ N] | 2.93 | A: GLU 224[ OE1] |
| 10 | C: ARG 102[ NH2] | 3.24 | A: VAL 280[ O] |
| 11 | C: TYR 308[ OH] | 2.85 | A: GLU 350[ OE2] |
| 12 | C: HIS 365[ NE2] | 2.54 | A: MET 360[ O] |
| 13 | C: SER 362[ OG] | 3.77 | A:PRO 364[ O] |
| 14 | C: ARG 258[ NH2] | 2.33 | A: LEU 417[ O] |
| 15 | C: SER  38[ O] | 3.83 | A: ALA  85[ N] |
| 16 | C: GLN 322[ O] | 2.69 | A: LYS 180[ NZ] |
| 17 | C: TYR 384[ OH] | 3.66 | A: LEU 214[ N] |
| 18 | C: GLN 311[ OE1] | 3.77 | A: SER 223[ N] |
| 19 | C: GLU  21[ OE1] | 2.97 | A: SER 223[ OG] |
| 20 | C: GLU 410[ OE1] | 3.76 | A: SER 240[ OG] |
| 21 | C: ARG 413[ O] | 3.30 | A: SER 243[ N] |
| 22 | C:ASP 392[ O] | 3.63 | A: LEU 265[ N] |
| 23 | C: ARG 390[ O] | 2.69 | A: GLY 267[ N] |
| 24 | C: MET 101[ O] | 3.86 | A: ARG 282[ NH1] |
| 25 | C: TYR 308[ OH] | 2.96 | A: ARG 282[ NH2] |
| 26 | C: LYS 105[ O] | 3.16 | A:ASP 351[ N] |
| 27 | C: SER 357[ O] | 3.82 | A: THR 366[ OG1] |

**Table S5** Prediction of molecular docking at hydrogen bond binding sites for RIPK3 and CASP6.

|  | **RIPK3** | **Dist.[A]** | **CASP6** |
| --- | --- | --- | --- |
| 1 | C: HIS 268[ NE2] | 3.51 | A:PRO 30[ O] |
| 2 | C: SER 202[ OG] | 3.32 | A: GLU 244[ OE1] |
| 3 | C: ARG 280[ NH2] | 3.87 | A: SER 254[ O] |
| 4 | C: ASN 272[ ND2] | 3.87 | A: ILE 282[ O] |
| 5 | C: ARG 196[ NH1] | 3.37 | A: THR 285[ OG1] |
| 6 | C:ASP 193[ OD2] | 3.11 | A: LYS 33[ NZ] |
| 7 | C: GLN 284[ OE1] | 2.61 | A: LEU 172[ N] |
| 8 | C:ASP 269[ OD1] | 3.57 | A: THR 243[ OG1] |
| 9 | C:PRO 270[ O] | 3.66 | A: THR 247[ OG1] |
| 10 | C: ARG 273[ O] | 3.34 | A: THR 247[ OG1] |
| 11 | C: CYS 129[ O] | 2.01 | A: GLN 255[ NE2] |
| 12 | C:ASP 278[ OD1] | 3.57 | A: PHE 273[ N] |
| 13 | C:ASP 269[ O] | 3.68 | A: LYS 277[ NZ] |
