# Supplementary material for: The assembly and activation of the PANoptosome promote porcine granulosa cell programmed cell death during follicular atresia
Source: J Anim Sci Biotechnol. 2024 Nov 5;15:147. doi: 10.1186/s40104-024-01107-3 (PMC11536665; doi:10.1186/s40104-024-01107-3)
Supplement: Supplementary file 2 — Additional file 2 Fig. S1 CASP6 cannot interact with RIPK1 and ZBP1.Fig. S2 CASP6 as a component of the PANoptosome. [file 40104_2024_1107_MOESM2_ESM.docx]

**Additional file 2**

**Fig. S1** CASP6 cannot interact with RIPK1 and ZBP1

**Fig. S2** CASP6 as a component of the PANoptosome

**Fig. S1** CASP6 cannot interact with RIPK1 and ZBP1
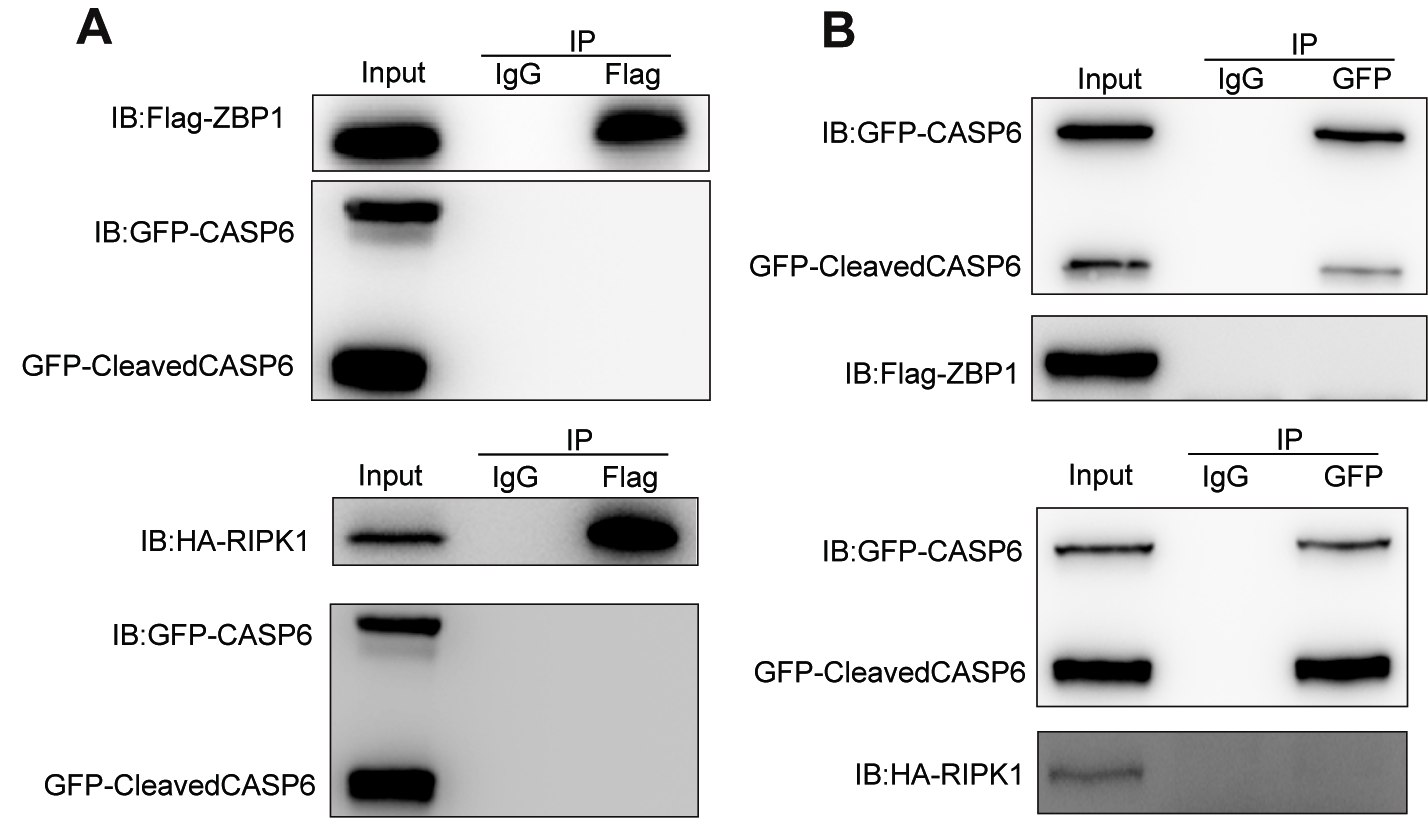
. **A** Flag-ZBP1 and GFP-CASP6 were co-transfected into 293T cells for 48 h for immunoprecipitation. **B** HA-RIPK1 and GFP-CASP6 were co-transfected into 293T cells for 48 h for immunoprecipitation


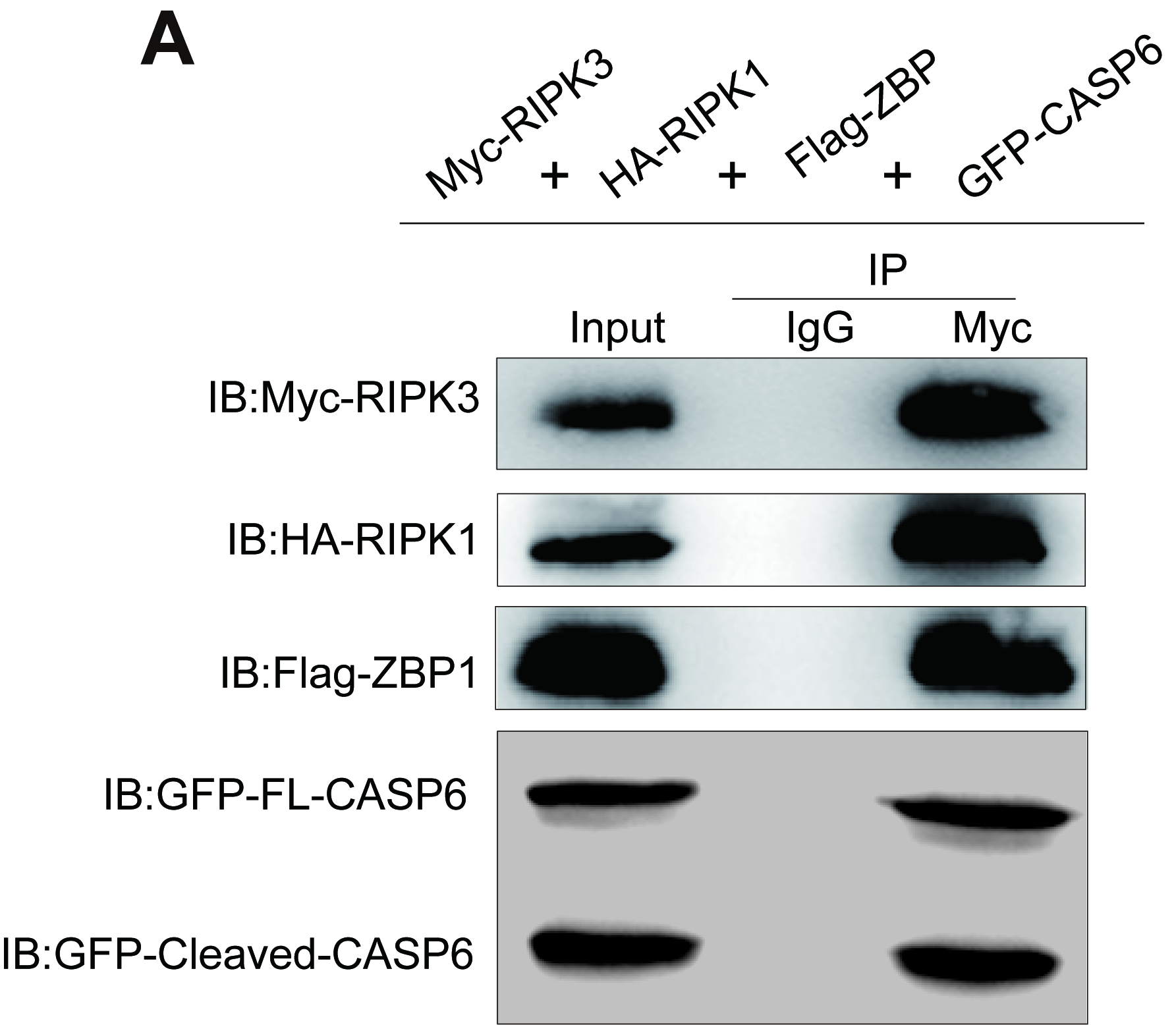


**Fig. S2** CASP6 as a component of the PANoptosome. **A** Flag-ZBP1, Myc-RIPK3, HA-RIPK1 and GFP-CASP6 were co-transfected into 293T cells for 48 h for immunoprecipitation
